# Haemodynamic Analysis of Branched Endografts for Complex Aortic Arch Repair

**DOI:** 10.3390/bioengineering9020045

**Published:** 2022-01-18

**Authors:** Sampad Sengupta, Mohamad Hamady, Xiao-Yun Xu

**Affiliations:** 1 Department of Chemical Engineering, Imperial College London, South Kensington Campus, London SW7 2AZ, UK; sampad.sengupta17@imperial.ac.uk; 2 Department of Surgery & Cancer, Imperial College London, St. Mary’s Campus, London W2 1NY, UK; m.hamady@imperial.ac.uk

**Keywords:** aortic arch, TEVAR, branched endograft, computational fluid dynamics, wall shear stress

## Abstract

This study aims to investigate the haemodynamic response induced by implantation of a double-branched endograft used in thoracic endovascular aortic repair (TEVAR) of the aortic arch. Anatomically realistic models were reconstructed from CT images obtained from patients who underwent TEVAR using the RelayPlus double-branched endograft implanted in the aortic arch. Two cases (Patient 1, Patient 2) were included here, both patients presented with type A aortic dissection before TEVAR. To examine the influence of inner tunnel branch diameters on localised flow patterns, three tunnel branch diameters were tested using the geometric model reconstructed for Patient 1. Pulsatile blood flow through the models was simulated by numerically solving the Navier–Stokes equations along with a transitional flow model. The physiological boundary conditions were imposed at the model inlet and outlets, while the wall was assumed to be rigid. Our simulation results showed that the double-branched endograft allowed for the sufficient perfusion of blood to the supra-aortic branches and restored flow patterns expected in normal aortas. The diameter of tunnel branches in the device plays a crucial role in the development of flow downstream of the branches and thus must be selected carefully based on the overall geometry of the vessel. Given the importance of wall shear stress in vascular remodelling and thrombus formation, longitudinal studies should be performed in the future in order to elucidate the role of tunnel branch diameters in long-term patency of the supra-aortic branches following TEVAR with the double-branched endograft.

## 1. Introduction

Pathologies in the aortic arch can arise due to a number of well documented causes: elevated blood pressure, trauma and congenital conditions, to name only a few. As is the case with most vascular conditions, pathological changes affect flow through the region and can compromise perfusion to distal parts of the body. Common aortic arch diseases include aneurysms and aortic dissections (AD) [1]. An aneurysm is a localised distention of the vessel wall, which results in an abnormal enlargement of the wall. Degenerative aneurysms in the arch are often asymptomatic and can go undetected. However, the increased use of diagnostic imaging has resulted in most aneurysms being detected and treated. If left untreated, this bulge in the wall can grow and eventually rupture with fatal consequences. An aortic dissection arises when there is a tear in the inner layer of the vessel wall and blood flows in between the layers of the wall, leading to the development of a ‘false lumen’ in the aorta alongside the actual ‘true lumen’. This can be further classified based on their location (known as the Stanford classification): Tears originating in the ascending aorta are referred to as type A aortic dissections (TAAD), whereas a tear in the descending aorta or extending into the abdominal region is referred to as a type B dissection. The incidence of aortic dissections is reportedly on the rise, with their incidence following major cardiac surgery estimated at 0.03–0.1%, with the highest risk being due to aortic valve replacement surgery [2]. TAAD occurring in the ascending aorta and aortic arch can significantly impair flow through the aortic arch branches which feed the brain and upper limbs, thereby increasing the likelihood of stroke. TAAD and arch aneurysm treatment poses unique challenges compared to other vascular pathologies primarily due to the location of the tear and the difficulty in treating the ascending aorta and aortic arch. 

The non-planar aortic arch with branches emerging from its outer curvature lends an added degree of complexity to the morphology of the region. Thus, aortic arch pathologies give rise to significant challenges to clinicians for treating these conditions, whether surgically or via endovascular means. Treatment methods have evolved over the years and have involved open surgical methods, hybrid techniques and endovascular means. Thoracic endovascular aortic repair (TEVAR) provide a minimally invasive treatment option with less post-operative time spent in hospital and quicker recoveries in patients [3,4,5]. Endovascular repair, initially developed for treating abdominal aortic aneurysms, has provided novel techniques for arch and ascending aorta treatment as well. Endografts used for aortic arch repair are designed to mimic a patient’s anatomy as closely as possible. The deployment and fixation of endografts coupled with the need for restoring the anatomical integrity of the region whilst avoiding invasive procedures is a challenge which clinicians are continually faced with [6,7]. The implantation of endografts in the arch, whilst crucial to the treatment procedure, can lead to alterations in flow patterns in the region. The double-branched endograft, which is the focus of this investigation, includes two inner tunnel branches that run along the wall of the device and lead into the innominate artery (IA) and left common carotid artery (LCCA), with the aim of providing sufficient perfusion of blood to the supra-aortic vessels [8,9,10]. 

Understanding the haemodynamic implications of endograft implantation is crucial to determining its potential long-term effects and post-intervention complications that may arise for the patient. Problems commonly associated with endografts include migration of the device, undesirable thrombus formation within the repaired region and endoleaks. Endoleaks arise when blood leaks into a gap between the endograft and vessel wall [11,12,13,14]. This can lead to the expansion of the true lumen or give rise to an aneurysm or compromise the integrity of the vessel wall even further. Current endograft designs aim at incorporating the flexibility of the arterial wall and improving fixation mechanisms, thereby minimising device migration and misalignment. Different endograft designs to combat aneurysms have been tested with an emphasis on preserving flow to the supra-aortic branches and investigating variations in surgical procedures involving the need for debranching or bypassing arch vessels. The positional stability of grafts is an important aspect of aortic repair and in vitro studies have been performed to find the balance between stabilising fixation forces and destabilising displacement forces experienced post-implantation. This also leads to predictions of possible device migration, misalignment and endoleaks by means of computational modelling [13,15,16,17].

Parameters of common interest in such studies include the flow velocity, pressure difference, wall shear stress and helicity. These are either averaged across or measured throughout various points of the cardiac cycle. It is often difficult to experimentally achieve a level of accuracy desirable in order to validate or be comparable to in vivo conditions; this is overcome by using computational tools that are designed to replicate in vivo conditions as closely as possible. Advances in imaging techniques and the integration with computational modelling allow for simulations to be carried out under physiologically realistic or patient-specific conditions [14,18]. The aim of this investigation is to evaluate flow in post-TEVAR aortas treated with a double-branched endograft. A particular area of interest is the effect of inner tunnel diameters on haemodynamics in the aortic arch and supra-aortic vessels and how varying the tunnel branch diameters alters the flow disturbance experienced in these regions. To this end, computational fluid dynamics (CFD) models are built to reproduce physiological conditions in the ascending aorta and aortic arch after the implantation of a double-branched endograft. The computational simulation results are used to provide a detailed analysis of the haemodynamics in the post-TEVAR aortas and to examine the effect of inner tunnel diameters on haemodynamics in the aortic arch and supra-aortic vessels.

## 2. Materials and Methods

### 2.1. Geometric Model

The aortic arch has complex anatomical features unique to every individual: namely, its curvature, non-planarity and major branches originating in the region. To fully capture these geometric complexities, geometric models were reconstructed from CT images obtained from specific patients. Two patients were included in this study and both presented with type A aortic dissection located in the arch region, underwent TEVAR using the RelayPlus double-branched endografts of the same tunnel branch diameter and neither required any further revascularisation. The overall characteristics of the two patients were thus largely identical, with the primary difference being the anatomy of the ascending aorta. This allowed for a comparison to be made of two cases undergoing the same treatment but with different anatomical features. Formal ethical approval was not required for this limited retrospective and anonymised study.

**Figure 1:**
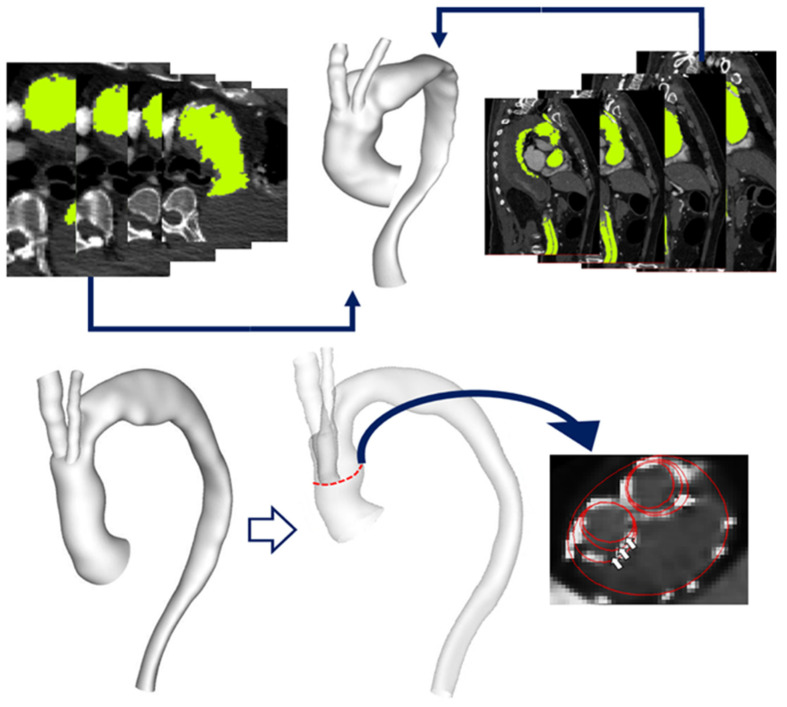
(**Top**) Multislice CT images used to generate anatomically accurate 3D reconstruction of the aortic arch. (**Bottom**) Reconstructed aorta model for Patient 1 with a transparent view indicating inner tunnel branches within the main endograft. The original inner tunnel branches are 12 mm in diameter and have been artificially modified to create models with branches of 10 mm and 8 mm diameter, respectively, as illustrated by the red outlines in a cross-sectional view of the ascending aorta, with the arrows indicating the three different branch diameters being investigated here.

The multislice images were analysed using a 3D image processing software, Mimics (v20, Materialise, Leuven, Belgium), to generate anatomically accurate 3D reconstructions of the patient’s vasculature, as shown in Figure 1. Segmentation techniques involving region growing methods were used to create parts which were then filtered out to isolate relevant regions of interest for each slice of the CT images. The reconstructed geometries were further smoothened using Meshmixer (v3.5 Autodesk Inc., San Rafael, CA, USA). The device being studied here is the Relay Plus double-branched endograft (Terumo Aortic), which consists of a main stent-graft (MSG) body and two branch stent-grafts (BSG’s) leading to the IA and LCCA. The BSG’s are attached to the MSG through two internal tunnels running along the inner lining of the MSG. The procedure involved the deployment of the MSG and insertion of BSGs leading into the IA and LCCA.

The reconstructed geometric models thus included the native ascending and descending aorta, the double-branched endograft implanted in the aortic arch and the supra-aortic vessels. Tunnel branches for one case (Patient 1) were artificially modified from their original dimensions (12 mm in diameter) to generate hypothetical models (1A and 1B) with tunnel branch diameters of 10 mm and 8 mm, respectively, in order to examine the effect of branch size on local flow (Figure 1). Unstructured meshes of approximately 8 million elements, with prismatic boundary layers applied at the walls, were generated using ANSYS ICEM (v15.0, ANSYS Inc., Canonsburg, PA, USA).

### 2.2. Mathematical Model and Boundary Conditions

Conservation of mass and momentum equations were used to describe 3D incompressible Newtonian pulsatile flow in the aorta, with a constant density of 1060 kgm^−3^ and dynamic viscosity of 0.004 Pa.s for the properties of blood. Although laminar assumptions are often made when simulating blood flow in the aorta, the complex nature of the geometry here and the combination of peak Reynolds number (Re) and Womersley number (Wo) based on the inlet diameter (Peak Re = 3451 and Wo = 23) led to the decision to employ a model capable of capturing laminar to turbulence transition. To this end, the SST-Tran (shear stress transport—transitional) model was adopted as it has been successfully applied to low Re transitional flow in arterial stenoses [19] and the human aorta [19,20]. Numerical solutions were obtained using ANSYS CFX (v15.0, ANSYS Inc., Canonsburg, PA, USA).

**Figure 2:**
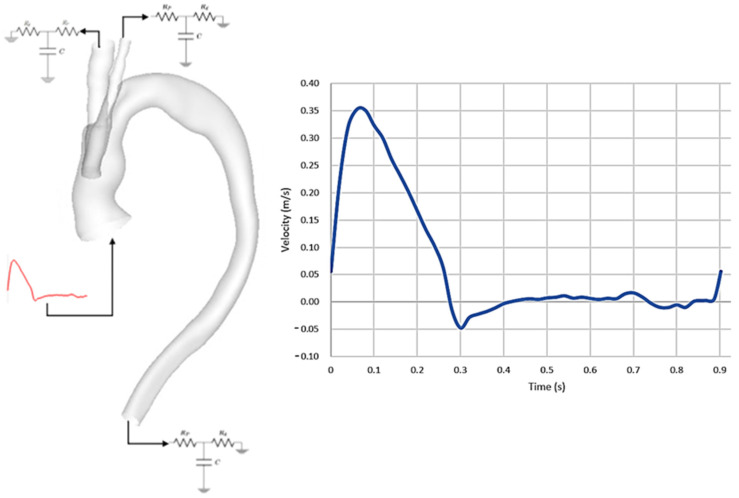
(**Left**) Representation of reconstructed 3D geometry showing prescribed boundary conditions, with inflow waveform imposed at the model inlet and 3−EWM prescribed at the three model outlets. (**Right**) Pulsatile velocity waveform prescribed at the inlet.

Physiological boundary conditions were imposed at the inlet and outlets (Figure 2); these included a 3-element Windkessel model (3-EWM) prescribed at the three outlets and a pulsatile inflow waveform used at the inlet. The inflow waveform was adapted from a previous study [21] along with the assumption of a flat velocity profile. A low inlet turbulence intensity (Tu) of 1% was prescribed to ensure the largely laminar nature of the flow was upheld and developing transitional/turbulent flow is modelled only if the need arises during the cardiac cycle [20,22]. The wall was assumed to be rigid with a no-slip boundary condition [23,24].

### 2.3. Haemodynamic Metrics

The focus of this study was to investigate the haemodynamic changes in the region of interest in response to the implanted endograft. A range of metrics were computed based on the obtained fluid velocity and pressure; these included wall shear stress (WSS) related metrics, displacement forces and helicity.

WSS is an important haemodynamic parameter owing to its association with the initiation and progression of arterial diseases [25,26]. It measures the tangential force exerted by the fluid on the vessel wall. Different WSS-derived parameters have been investigated here: Time-averaged WSS (TAWSS) and Transverse WSS (TransWSS). TAWSS is the average of the WSS magnitude over the cardiac cycle and can be defined as:

(1a) $$\mathrm{TAWSS}=\frac{1}{T}\int_{0}^{T}\left|\tau_{w}\;\right|dt$$

where *T* is the time period of a cardiac cycle, and ***τ_w_*** is the wall shear stress vector. TransWSS is defined as the “average over the cardiac cycle of WSS components perpendicular to the temporal mean WSS vector, with which endothelial cells are assumed to align” [27,28,29]. It can thus be used to quantify deviations in the direction of WSS vectors throughout the cycle, a phenomenon which demonstrates the multidirectionality and oscillations in flow and can be expressed as:

(1b) $$\mathrm{TransWSS}=\frac{1}{T}\int_{0}^{T}\left|\tau_{w}\cdot \left(n\times \frac{\int_{0}^{T}\tau_{w}dt}{\left|\int_{0}^{T}\tau_{w}dt\right|}\right)\right|dt$$

To describe vortical flow structures in the aorta, the *λ*_2_ criterion for incompressible flows was evaluated and displayed as isosurfaces running through the fluid domain [30,31,32]. The *λ*_2_ criterion can be expressed as:

(2) $$\lambda_{2}=\frac{\partial v_{x}}{\partial y}\frac{\partial v_{y}}{\partial x}+{\left(\frac{\partial v_{y}}{\partial y}\right)}^{2}+\frac{\partial v_{y}}{\partial z}\frac{\partial v_{z}}{\partial y}$$

where *λ*_2_ is the second eigenvalue of the tensor ***S^2^ + Ω^2^***, with ***S*** and ***Ω*** being the symmetric and antisymmetric parts of the velocity gradient tensor, respectively. ***V_x_***, ***v_y_***, ***v_z_*** are the velocity components in the *x*, *y*, and *z* directions, respectively. Another measure of complex flow structures in the aorta is helicity, which can be quantified using a synthetic descriptor, namely, helical flow index (HFI) [33,34,35]. HFI can be calculated from local normalised helicity (LNH), which is defined as:

(3a) $$\mathrm{LNH}\;\left(s,\;t\right)=\frac{V\left(s,\;t\right)\;\cdot \;\omega \left(s,\;t\right)}{\left|V\left(s,\;t\right)\right|\left|\omega \left(s,\;t\right)\right|}$$

LNH is a function of space (***s***) and time (*t*), with V(s, t) and ω(s, t) being the velocity field and vorticity field, respectively, of the fluid domain. HFI is then computed from LNH using a set number of particles (*N_p_*) released at the model inlet and following the trajectory of each particle within a set time interval. Considering the path of an individual particle *k*, the helical flow index can be represented as:

(3b) $$hfi_{k}=\frac{1}{N_{k}}\overset{N_{k}}{\underset{j=1}{\sum }}{\left|\mathrm{LNH}\right|}_{j}$$

where *N_k_* is the number of points *j* (*j* = 1, … *N_k_*) in the *k*th trajectory in which LNH has been calculated. Combining this for all particles *N_p_* in the fluid domain, HFI is calculated as:

(3c) $$\mathrm{HFI}=\frac{1}{N_{p}}\overset{N_{p}}{\underset{k=1}{\sum }}hfi_{k}$$

This yields HFI ranging from 0 to 1, with the value for normal aortic flow being reported to be between 0.3 and 0.5 [34,35,36].

## 3. Results

### 3.1. Flow Patterns and Pressure

**Figure 3:**
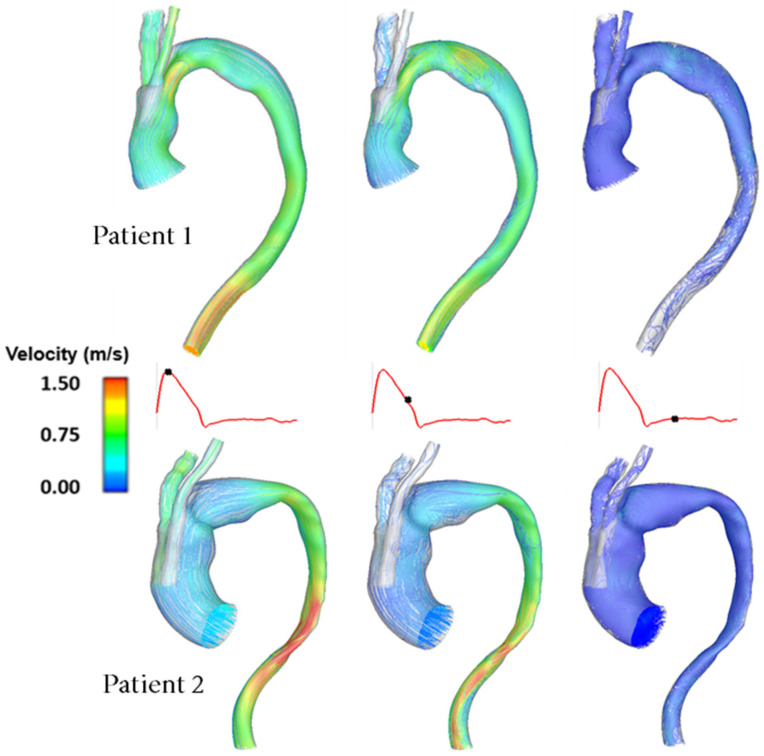
Comparison of instantaneous velocity streamlines obtained for Patient 1 and Patient 2 at three characteristic time points in the cardiac cycle.

In order to illustrate the dynamics of the pulsatile flow of blood through the vessels, instantaneous velocity streamlines are plotted and displayed at three distinct points in the cardiac cycle: peak systole, mid-systolic deceleration and mid-diastole, as shown in Figure 3. This allows for the observation of the general flow features and qualitative comparisons between the two patient-specific models. Clearly, there are considerable geometric differences between the two patients, resulting in dramatically different flow patterns in the aorta.

**Figure 4:**
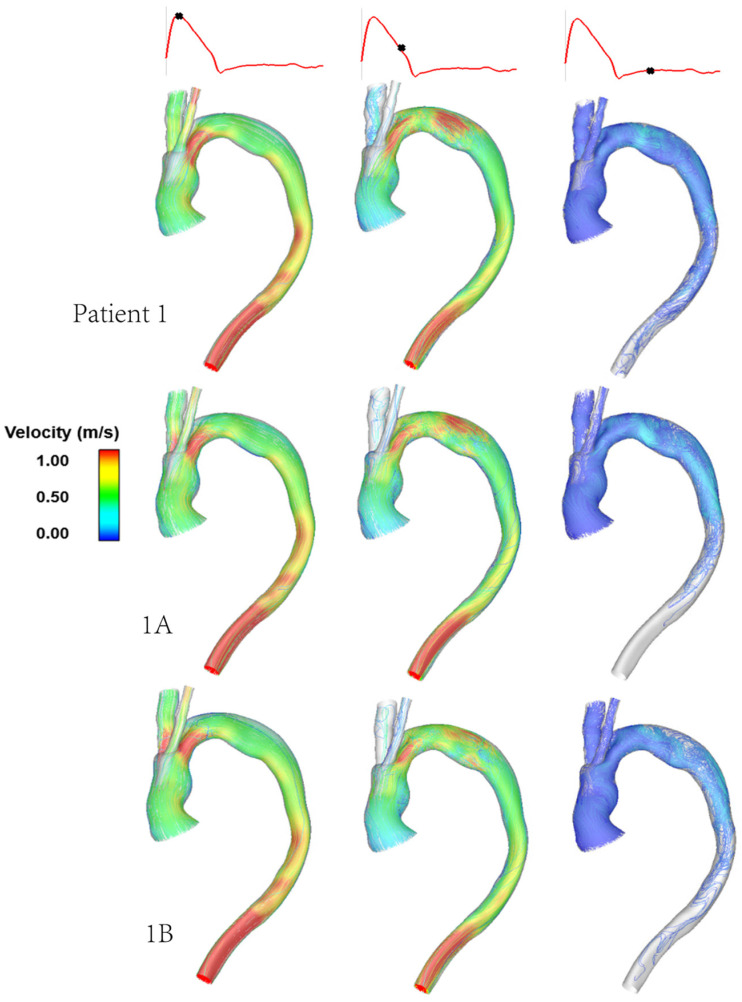
Comparison of instantaneous velocity streamlines obtained for cases of different diameters of inner tunnel branches for the same patient geometry, at three characteristic time points in the cardiac cycle. Patient 1 (**top** row), model 1A (**middle** row) and model 1B (**bottom** row) have inner tunnel diameters of 12 mm, 10 mm and 8 mm, respectively.

**Figure 5:**
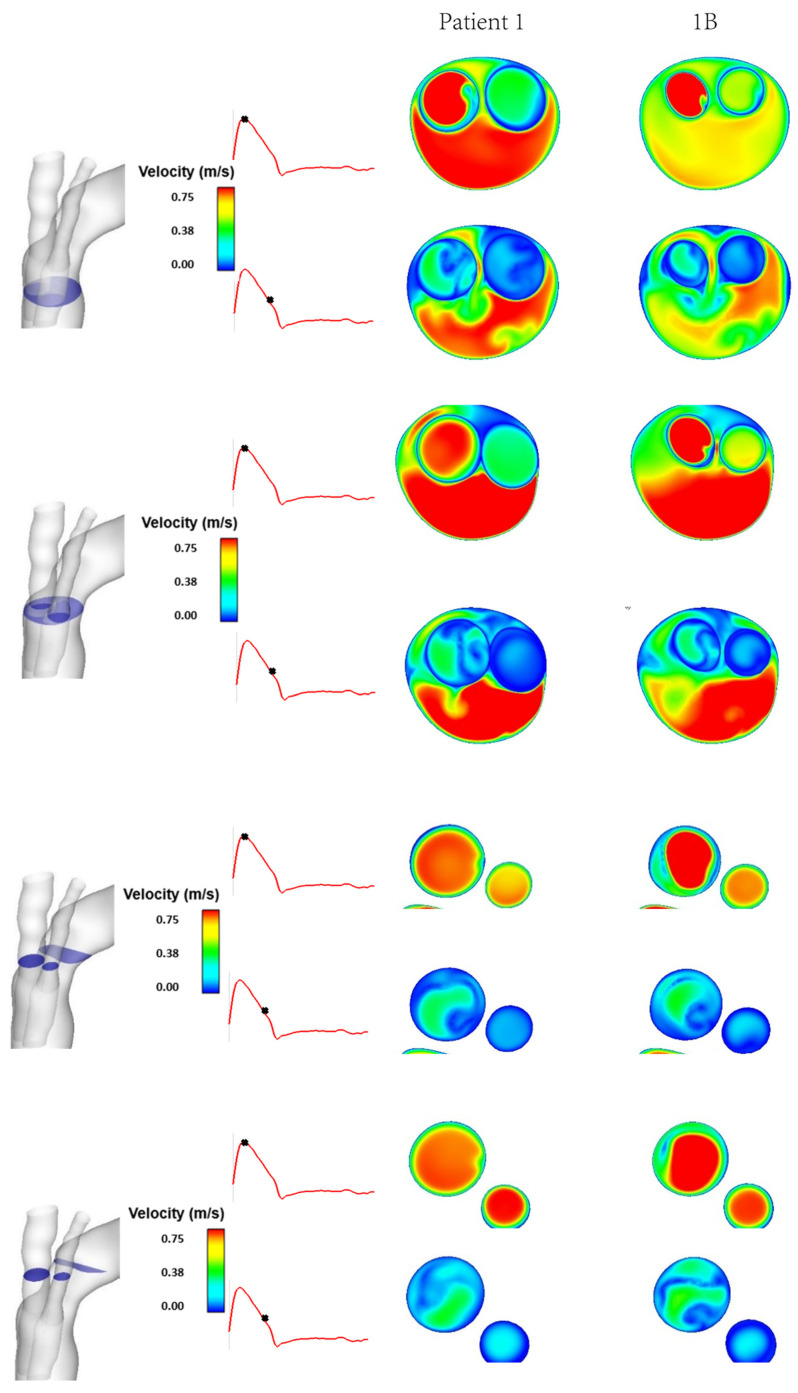
Comparison between Patient 1 and model 1B of instantaneous velocity magnitudes at different cross-sectional planes at peak systole and mid-systolic deceleration phases of the cardiac cycle.

Figure 4 shows the instantaneous velocity streamlines for cases 1, 1A and 1B, which have tunnel branch diameters of 12 mm, 10 mm and 8 mm, respectively. This demonstrates changes in the magnitude of velocity for different tunnel branch diameters. Narrower tunnel branches tend to cause accelerated flow into the larger emerging arch branches. The closer the diameter of tunnel branches to the supra-aortic branch that it leads to, the smoother the flow appears to be. This can be visualised from a different perspective in Figure 5, which depicts the velocity magnitude at cross-sectional planes taken at different locations in the ascending aorta and emerging branches. By comparing cases 1 and 1B, which have 12 mm and 8 mm branches, respectively, it can be clearly seen that the larger tunnel branch leads to a smoother perfusion of flow to the IA, without any local acceleration or deceleration in flow velocity. Similarly, the 8 mm tunnel branches in case 1B smoothly guides the flow to the LCCA branch, both having a similar diameter. The streamlines in Figure 4 also reveal subtle changes in flow pattern in the aortic arch, immediately after the emergence of the supra-aortic branches. Their effects on local WSS will be explored later. 

**Figure 6:**
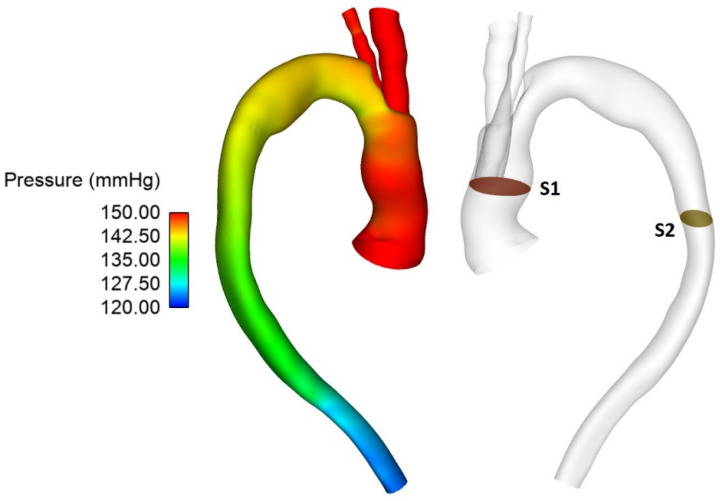
Pressure distribution throughout the vessel at peak systole for Patient 1 (left) with the pressure difference measured between planes S1 and S2 (right).

In addition to observing the flow patterns in the region of interest, the pressure distribution along the aorta was examined (Figure 6) and pressures at the proximal (plane S1) and distal end (S2) of the endograft were evaluated. These were then used to calculate the difference in pressure upstream and downstream of the endograft for cases 1, 1A and 1B in order to determine the effect of tunnel branch diameters on pressure drop through the grafted region (Table 1).

### 3.2. Wall Shear Stress

**Figure 7:**
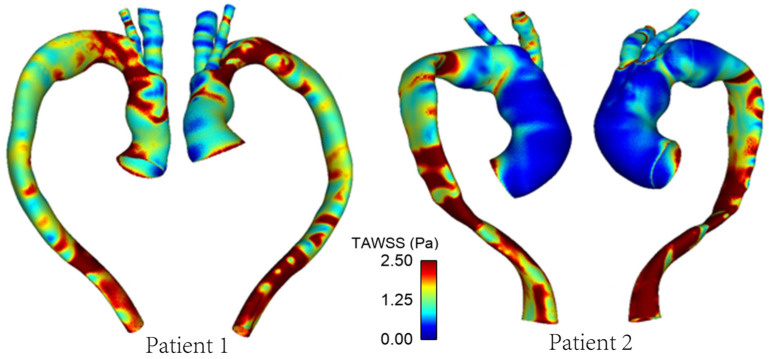
TAWSS distribution in the repaired aortas of Patients 1 and 2.

As mentioned previously, WSS serves as an important metric in analysing aortic flow and different WSS plots have been presented here. Figure 7 shows a comparison of time-averaged WSS (TAWSS) in the repaired aortas of Patient 1 and Patient 2. The most obvious difference between the two patients is observed in the ascending aorta, with much lower values of TAWSS in Patient 2 due to the larger lumen diameter compared to the corresponding sections of Patient 1.

**Figure 8:**
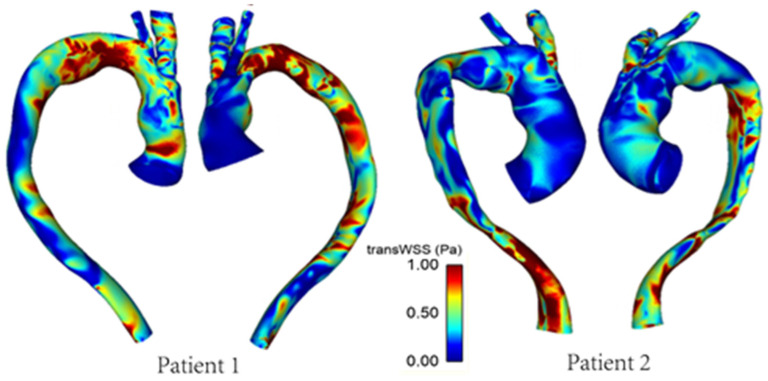
TransWSS distribution in the repaired aortas of Patients 1 and 2.

Figure 8 shows the differences in transverse WSS (transWSS) between Patient 1 and Patient 2. TransWSS, as opposed to TAWSS, takes into account the multidirectionality of flow in the region. It can be used to quantify deviations in the direction of WSS vectors throughout the cardiac cycle, hence demonstrating the multidirectional nature and oscillations in the flow.

**Figure 9:**
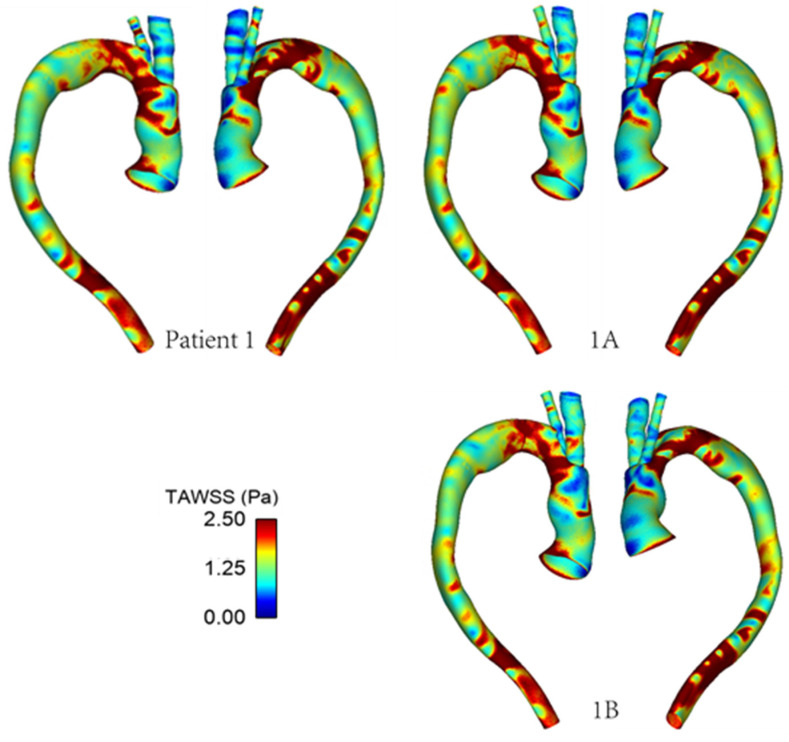
TAWSS distribution in the repaired aorta of Patient 1 with different tunnel branch diameters (1–12 mm, 1A–10 mm, 1B–8 mm).

**Figure 10:**
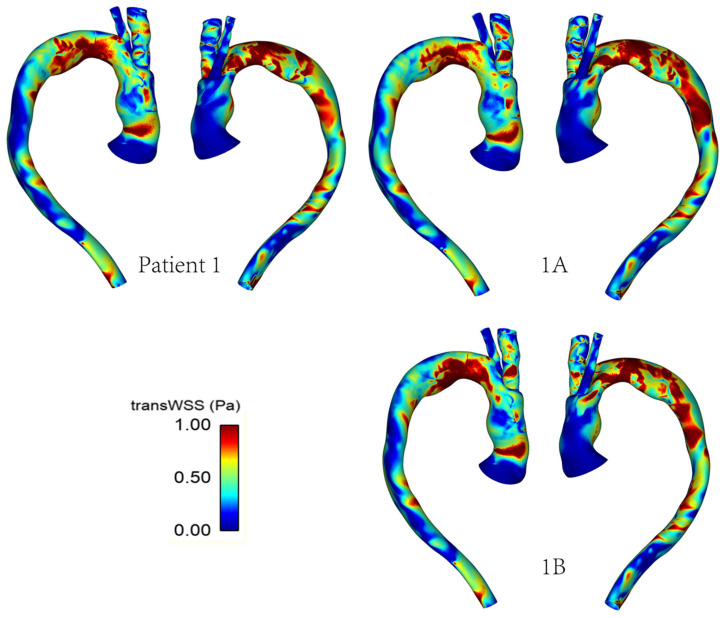
TransWSS distribution in the repaired aorta of Patient 1 with different tunnel branch diameters (1–12 mm, 1A–10 mm, 1B–8 mm).

Figure 9 and Figure 10 show the TAWSS and transWSS in the repaired aorta of Patient 1 and its artificially modified variants models 1A and 1B. The spatial distribution of TAWSS remains qualitatively similar in all cases, with negligible quantitative differences for different tunnel branch diameters. However, variations in the spatial distribution of transWSS can be noted in Figure 10, indicating that varying the tunnel branch diameters primarily affect the flow disturbance and multidirectional flow rather than the magnitude of WSS throughout the cardiac cycle.

### 3.3. Vortical and Helical Flow

**Figure 11:**
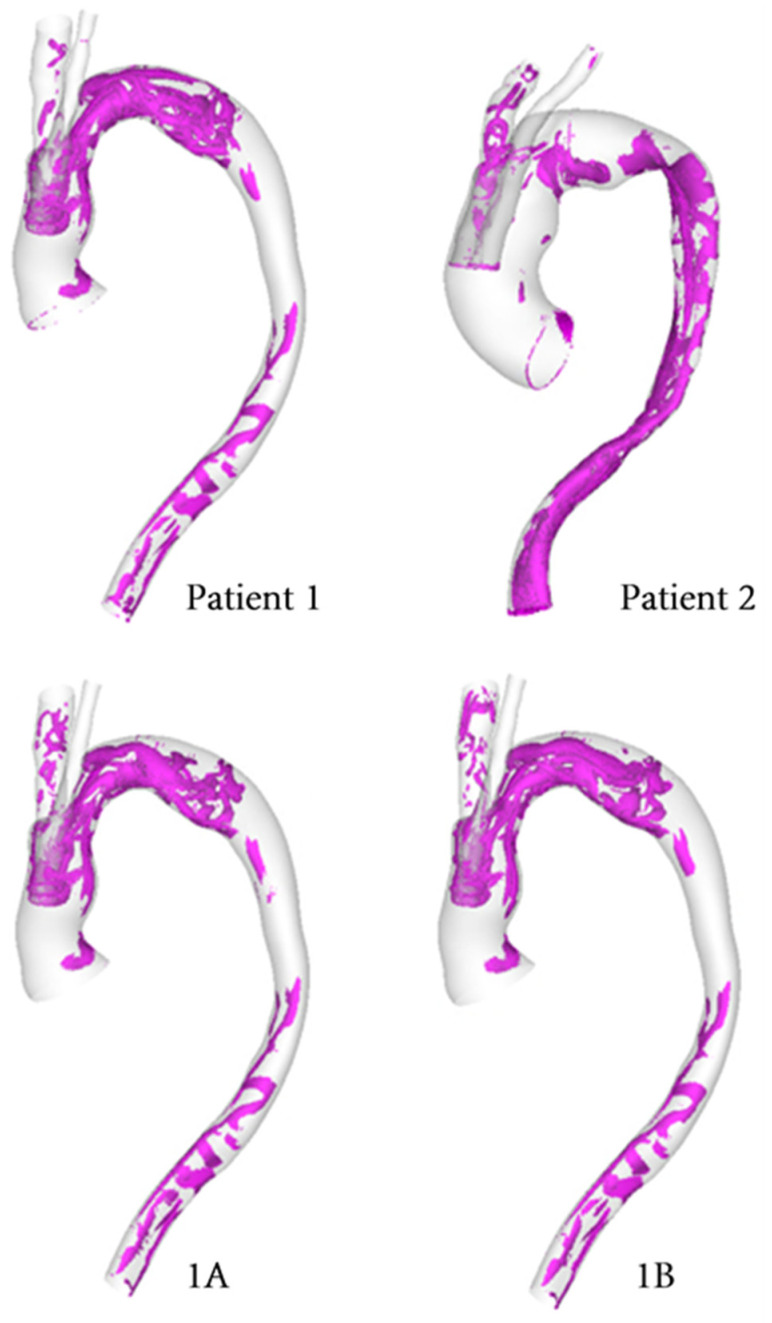
Isosurfaces used to depict vortical structures isolated using the *λ*_2_ criterion computed for each model at mid-systolic deceleration. (1A–10 mm, 1B–8 mm).

The vortical flow structure can be visualised by using the λ_2_ criterion as shown in Figure 11. The λ_2_ threshold was adjusted in order to properly isolate the relevant vortical flow through the vessel and the same has been used for all models for comparison. Figure 11 highlights the effect of geometry on the development of vortical flow, with Patient 1 and Patient 2 showing different vortical flow features along the aorta. Differences among the various cases for Patient 1 are small and are confined to the arch immediately downstream of the emerging arch branches. In addition, the helical flow index (HFI) was calculated for all cases, and the results are summarized in Table 2.

## 4. Discussion

The complexities posed by aortic arch repair have brought about the need for branched endografts to be used in TEVAR. The RelayPlus device was specifically designed to accommodate the supra-aortic branches, with inner tunnels built within the main stent-graft body to allow insertion of the branch stent-grafts. This investigation entails a detailed haemodynamic analysis of the device by carrying out physiologically realistic computational modelling.

The configuration of the RelayPlus device requires the coverage of the left subclavian artery (LSA), but revascularisation was not deemed necessary for the two patients included in this study. It has been shown that sufficient perfusion to the cerebral arteries can be maintained following LSA coverage [37]. The tunnel branches in the RelayPlus device serve the purpose of guiding flow into the IA and LCCA, ensuring the perfusion of blood to the supra-aortic vessels. This is crucial as the supra-aortic branches carry blood to the upper limbs and, most importantly, the brain [38,39]. The disruption of cerebral perfusion can occur as a result of occlusions in the aortic arch branches, improper fixation of endografts during TEVAR or a lack of flow due to leaks or thrombosis in the region [40,41,42,43].

### 4.1. Patient Comparison

Patient 1 and Patient 2 presented with similar aortic arch dissections which were treated with TEVAR using the same double-branched endograft, despite having different anatomical features and dimensions. This allowed us to examine the performance of the device in various anatomical conditions. A common trend in both cases is flow acceleration in regions where the lumen is narrowed. As can be seen in Figure 3, there is a higher velocity flow in the ascending aorta of Patient 1 compared to Patient 2. Although both cases have the same tunnel branch diameters of 12 mm, Patient 1 has a smaller ascending aorta diameter, hence, the tunnel branches occupy a large proportion of the ascending aorta cross-section (Table 2). Accelerated flow is also observed in the proximal descending aorta of Patient 2 where the lumen narrows considerably. In addition, some degree of recirculating flow is present in the arch region in mid-diastolic deceleration, which is attributed to local geometric variations in the arch introduced by the window in the superior portion of the endograft. In Patient 1, the proximal arch is narrowed which is followed by a small bulge, while the opposite is true for Patient 2.

The helical flow experienced in the aorta arises due to the non-planar nature of the vessel. Torsion induced helical flow may stabilise the flow of blood in the aorta, reducing flow disturbance and suppressing the separation of flow [35,36,44,45,46]. The flow topology changes throughout the cardiac cycle, thereby altering the helical nature of the flow in the system due to a local exchange of writhe and twist helicity in the interacting vortical tube strands. This can be noted by changes in vortical flow patterns throughout the cycle with the most prominent structures occurring in the mid-systolic deceleration phase. This is comparable to the helical flow pattern observed in healthy aortas [34,35]. As revealed by using the λ_2_ vortex identification criterion (Figure 11), Patient 1 has a greater degree of vortical structures in the aortic arch compared to Patient 2. This can be attributed to the narrowing of the lumen in the proximal arch, where the window for the emerging BSGs is located.

### 4.2. Effect of Tunnel Branch Diameter

Figure 4 compares the aortic flow pattern in Patient 1 with those in models 1A and 1B, which have the same aorta geometry but varying tunnel branch diameters of 12 mm, 10 mm and 8 mm respectively. The branch dimensions are based on those available for use as BSGs in the Relay Plus device. Our results show that reducing the tunnel branch diameter causes local flow acceleration into the IA. This is more obvious in model 1B, where there is a jet of higher velocity flow in the emerging IA branch at peak systole. Closer examination of the flow patterns in the branches (Figure 5) also reveal that flow in the IA is less uniform in model 1B compared to Patient 1. This is because the 8 mm tunnel branch in model 1B is much narrower than the diameter of IA, resulting in an expansion in cross-sectional area as flow emerges from the inner tunnel. A sudden expansion combined with a change in flow direction due to the IA take-off angle from the arch cause the high velocity flow to be skewed towards one side of the vessel wall with low velocity flow on the opposite side. It is interesting to note that the effect of changing tunnel branch diameter on flow in the LCCA is less obvious, although there appears to be a slightly smoother transition of flow to the LCCA with the 8 mm tunnel branch, which is closer to the LCCA diameter. Both the 12 mm and 10 mm tunnel branches are larger than the ICCA diameter in Patient 1, creating a converging passage as flow is directed into the ICCA.

Compared to Patient 1, models 1A and 1B also show subtle changes in the flow pattern in the aortic arch immediately after the window for BSGs. This is because a larger tunnel branch diameter results in a larger proportion of the aortic lumen being taken up by the branches: 32% in Patient 1, 22% in model 1A and 14% in model 1B. Flow acceleration through the narrowed lumen of the arch contributes to increased multidirectional flow in the region, giving rise to increased spatial variation in WSS, as can be seen in Figure 9. WSS is known to play an important role in vascular remodelling and thrombosis, with low and oscillating WSS corresponding to regions of thrombus formation and intimal thickening. Whilst this may be desirable in certain cases post-TEVAR, for example, to promote false lumen thrombosis in aortic dissections, low and oscillatory WSS can increase the risk of thrombus formation in the tunnel branches. The indices presented here are time-averaged WSS (TAWSS) and transverse WSS (transWSS): TAWSS conveys the spatial distribution of WSS magnitude averaged over the cardiac cycle, whilst transWSS portrays the multidirectional nature of WSS. Figure 9 and Figure 10 show the influence of tunnel branch diameters on these WSS metrics. While changing the tunnel branch diameters do not appear to have altered the TAWSS pattern in the arch, there are notable differences in the arch vessels, especially in the proximal portion of the IA.

The non-planar nature of the aorta leads to the formation of vortical structures in the vessel, with complex helical flow patterns present throughout the arch and progressing down the descending aorta. Such patterns can often be difficult to isolate and quantify, which prompts the use of the λ_2_ criterion as a means of generating isosurfaces in order to better identify the vortex cores developing in the region. The vortical structure of flow observed via the λ_2_ isosurfaces tends to vary with the tunnel branch diameters (Figure 11), where isosurfaces near the wall tend to coincide with regions of high transWSS, which is due to changes in the direction of flow near the vessel wall. Furthermore, the helical flow index (HFI) computed here serves as an objective and reliably quantitative means for evaluating the helical nature of flow path. The values for the HFI for all cases considered here lie within the range (0.3–0.5) expected in a normal aorta [35].

Overall, our simulation results show that post-TEVAR aortic haemodynamics is close to normal for the two patients examined here. Both patients had aortic arch dissection following partial TAAD repair. The implantation of the double-branched endograft successfully covered the main entry tears and induced false lumen thrombosis, as observed in the CT images used to reconstruct the geometric models. The double-branched device also provides a suitable conduit to guide flow into the supra-aortic branches, thereby ensuring sufficient cerebral perfusion. In order to assess the risk of device migration, flow-induced displacement forces acting on the main stent-graft body should be evaluated. Based on a previous computational study of a similar double-branched device, the maximum displacement forces were around 22 N, which is well below the threshold value to dislocate a nonplanar stent-graft [47]. The unique fixation method of the RelayPlus device at the landing zone also helps to reduce the risk of stent-graft misalignment or displacement post-implantation. The plow patterns and the WSS metrics in the arch branches also indicate that there is no major risk of thrombus formation within the branches, but the diameter of the inner tunnel branch leading to the IA should be carefully chosen as a small tunnel branch may induce local flow disturbance in the proximal portion of the IA. From the fluid dynamics point of view, the tunnel branch diameters should be as close as possible to the actual diameters of the corresponding arch branches in order to allow for a smooth transition of flow into the supra-aortic pathway. Differences in diameter between the inner tunnel branches of the main stent-graft and the respective branches lead to a diverging or converging passage as flow enters the IA and LCCA. It is known that a divergent configuration or a sudden expansion tends to destablise flow and hence should be avoided as much as possible to ensure the long-term patency of the supra-aortic branches.

The simulations were carried out by assuming the aortic wall to be rigid and thus the effect of aortic wall compliance was not considered in this study. The computational model can be further improved by incorporating wall distensibility and aortic root motion along with inflow waveforms specific to the patient (which were not available for the present study).

## 5. Conclusions

A comprehensive haemodynamic analysis was carried out on two patient cases where the double-branched RelayPlus endografts were used in TEVAR to treat aortic arch dissections. Our patient-specific simulation results allowed us to conclude that normal aortic flow patterns were restored in both patients following the TEVAR procedure. Our analysis and comparisons between different tunnel branch diameters further demonstrated the influence of tunnel diameters on localised flow patterns and WSS distribution, with smaller tunnel diameters causing more disturbance when the flow transitioned to the supra-aortic branches. This finding has important implications for predicting and maintaining the long-term patency of the supra-aortic branches following implantation of the double-branched endograft. In order to minimise disturbed multidirectional flow in these branches, thereby mitigating the risk for thrombus formation, the tunnel branch diameters should be as close to the respective supra-aortic branch diameters as possible. Future longitudinal studies are needed to provide clinical evidence to validate these findings.

## Figures and Tables

**Table 1:** Pressure drop across the endograft for different tunnel branch diameters.

Model	Tunnel Branch Diameter (mm)	Δ Pressure (mmHg)
Patient 1	12	10.45
1A	10	9.95
1B	8	9.53

**Table 2:** Geometric dimensions and helical flow index (HFI) for different models, where Patient 1 and Patient 2 have different overall geometries, while Patient 1, models 1A and 1B have the same aorta geometry but different tunnel branch diameters. C.S. stands for cross-sectional.

Model	Tunnel Branch Diameter (mm)	Lumen C.S. Area at Tunnel Branch Mouth (mm^2^)	% of Lumen C.S. Area Taken up by Tunnel Branches	HFI
Patient 1	12	706	32.03	0.391
Model 1A	10	706	22.25	0.380
Model 1B	8	706	14.24	0.397
Patient 2	12	1219	18.55	0.476

## Data Availability

The data presented in this study are available on request from the corresponding author. The patient data are not publicly available due to restrictions.
